# Transition of Mental Health to a More Responsible Service in Iran

**Published:** 2017-01

**Authors:** Behzad Damari, Siamak Alikhani, Sahand Riazi-Isfahani, Ahmad Hajebi

**Affiliations:** 1 National Institute of Health Research, Tehran University of Medical Sciences, Tehran, Iran.; 2 Health Policy Researcher, Ministry of Health and Medical Education, Tehran, Iran.; 3 Research Center for Addiction and Risky Behavior (ReCARB), Psychiatric Department, Iran University of Medical Sciences, Tehran, Iran.

**Keywords:** *Mental Health*, *Primary Health Care*, *Transition*

## Abstract

**Objective: **This study proposed a model for provision of an effective universal coverage for mental health services based on global and national experiences, available resources and the nature of primary health care system of Iran to reduce the burden of mental health conditions.

**Method:** A framework with prioritized mental and social health services was devised through a review of literature and policy documents. It was then adapted using inputs from the stakeholders and experts.

**Results:** The new model included 2 basic and specialized service strata: a PHC-based infrastructure and essential requirements needed to establish the service. Our proposed socio-mental health approach is based on a WHO recommendation.

**Conclusion**: The key features of the model, which is going to be tested in a pilot study in 2015, are setting up a system for organized referrals to specialized mental facilities and compatibility with the existing primary health care system. Moreover, to achieve this goal, socio-mental health technicians should be employed.

According to the World Health Organization (WHO), “Mental health is defined as a state of well-being in which every individual realizes his or her own potential, can cope with the normal stresses of life, can work productively and fruitfully and is able to make a contribution to her or his community”(1).

It is estimated that 919 million people suffer from mental disorders worldwide, accounting for about 19% of the adult population (2). Mental disorders including drug abuse are responsible for 14% of the disease burden; of this, 75% occurs in low- and meddle- income counties (3). Studies conducted in 1999, 2001 and 2011 determined the prevalence of psychological disorders in Iran to be 21%, 17.10%, 23.6%, respectively (7). These rates are significantly high compared with the global statistics (7). Mental and behavioral disorders ranked second for burden of disease after intentional and unintentional accidents in 2003 (8). The burden of mental and behavioral disorders was estimated to be 41% of the total disease burden in 2005 (9). 

Despite the high prevalence of mental disorders, mental health services do not receive enough attention.

According to WHO, only 60% of countries have policies, 71% have programs and 59% have laws and regulations for mental health (10).

With respect to care provision, a community-based health service provided by professionals other than health workers is a preferred way to increase the accessibility of services (11). This is especially useful for low- and middle- income countries (10) where the health systems usually faces difficulties with the attitudes of the public, continuity and comprehensiveness of the services (3). That is why a more concrete understanding of the process of integrating these services must be in place before commencement of any services (12).

There are 3 main strata of obstacles on the path of an effective mental health intervention:

1. At the policy-making level: Insufficient financing, lack or inadequacy of mental health legislations and regulations and discrimination committed by health insurances against individuals with mental and behavioral disorders

1. Social stigma and discrimination, war and conflict, disasters and poverty
2. At the health system level

Moreover, societal matters have synergistic effects on the onset, persistence and severity of mental disorders (13). Therefore, integrating social services with mental health care is very beneficial (14). The aim of social services is to encourage an independent life for an incapable individual and provide financial support for those who need help to live independently (15). Being familiar with the social status, employment, working environment and the physical and social environment of the community members makes a social worker an asset for any health care team (16).

In 2012, Allen criticized multi-professionalism of medicine and suggested setting up a center called “medical home” instead of specialized clinic in which multidimensional primary care is provided by physicians. Social workers are the key players in the proposed establishment because their occupation entails at least 5 major characteristics: 1) patient-centered, 2) comprehensive, 3) coordinated, 4) provide superb access to care and 5) have a system-based approach to quality and safety (12).

Iran has been experiencing urbanization in the past decades; its urban population composed of 71% of the total in 2011 (17). It is estimated that more than 75% of the outpatient mental health services be provided by private clinics and offices of psychologists and psychiatrists. The public sector provides the majority of in-patient services (19). Moreover, there is not a well-defined public-private partnership mechanism (18). As a result, psychological disorders often go undiagnosed and untreated (19).

The new Mental Health Promotion Program of Iran was established in 2012 (20). This plan offers 3 basic strategies to meet the goals:

1. Qualitative and quantitative improvement of mental health services
2. Enhancing mental health literacy of different social groups
3. Investment in decreasing mental health risk factors

Implementing these strategies raise the need to revise the existing mental health care system. The aim of this study was to redesign the current mental health service through the primary health care system.

## Materials and Methods

The first stage of this study was a situation analysis of mental health services followed by a stakeholder analysis and consensus building to propose a new system using a qualitative approach. 

The information was gathered from 3 sources: 1) reviewing documents, 2) stakeholders and experts opinions and 3) group discussions.

The following categories of documents were used in this survey:

1. Studies on mental health services in Iran (21-23)
2. High-level documents (24)
3. Status of mental health resources in Iran (budget, workforce, observatory and health facilities)
4. Determinants of social and mental health in Iran (health system objectives) from routine data (24-26)
5. WHO recommendations

The study was conducted in 3 phases:

Using a directed approach content analysis, the data were collected based on the conceptual model:

1. By reviewing the documents, a framework with prioritized social and mental services was devised. All components of a system were considered and the external and internal environments were identified to design the framework. Moreover, a questionnaire was developed and used to collect stakeholders’ opinions.
2. Stakeholders were addressed according to their power and influences using a stakeholder analysis (Table 1). Intentional sampling method was used to recruit the experts. They were selected from the specialists of public health, health care management, psychology, psychiatry and social work. Structured interviews were conducted using a set of open-ended questions. The tool and the conceptual framework were sent to the interviewees by email and then the face-to-face interview was arranged. All interviews were recorded with the permission of the interviewees and transcribed by a single interviewer. The interviewer was a trained researcher and familiar with the in-depth interview method. A thematic analysis was conducted following the conceptual model. Scientific rigor of the result was confirmed using credibility, dependability, confirmability and transferability. Credibility of the interviews was assessed by rechecking the contents with each interviewee. Dependability, confirmability and transferability were confirmed in a group discussion session with all interviewees and the research team.
3. A draft of the mental health services charter was developed. The framework of the charter was based on the objectives, structure, routines and standards, management style and resources. Then the charter was reviewed and criticized by experts in 2 focus group discussions. Eventually, the final charter was designed in 2 sections:

- Regulations for implementation covering the concepts and regulate documents, objectives, services, implementation structure, resources and management style
- The operational guidelines

## Results

We reviewed all available published and unpublished papers and documents related to mental health initiatives in Iran. Here is a summary of our findings:

1. The first mental health program of Iran was designed in 1988 and received endorsement from the Ministry of Health in 1990 (22). It was a pilot, tested from 1992 to 1994 and after more than 2 decades, the program now covers 18 million (82.8%) rural residents and 10 million (21.7%) citizens in urban areas. The program mainly addresses severe mental health disorders, epilepsy and mental retardation. In the basic health care unit in rural areas, a community health worker is responsible for providing essential mental health services including training, case finding, referrals to a physician and handling follow-up visits. The next level of care in villages is rural health center in which a general practitioner is responsible for treatment and referrals to specialists. The same setting is established for urban areas; however, the coverage of urban health centers is not as comprehensive as villages, especially in larger cities. General practitioners treat 80% of the patients visiting rural health centers and refer about 20% of the cases to a specialist. While the program has been proven successful for villages, it is not sufficient for the urban areas (22).
2. Another model for urban mental health services has been designed based on a national policy of transition to a family physician approach, but has not yet been implemented (27). The family physician program is now in the pilot phase.
3. The experience of community-based mental health centers (CMHC): The structure of CMHC for urban areas was designed to target the followings: 1) those with neurotic disorders including depression and anxiety and 2) patients with severe mental disorders including schizophrenia, bipolar disorder and suicide attempters. General practitioners and case managers provided services for neurotic patients through a collaborative care method. They cooperated to reach the goal of early diagnosis and treating depression and anxiety disorders under the CMHC initiative. There were services such as home visits and telephone follow-ups by expert teams as well. It was recommended that the program be studied to assess its cost-effectiveness at the next phase (28).
4. The State Welfare Organization, the main provider of social services in Iran, targets mental health as well. The organization emphasizes the empowerment of individuals and families and improving mental health in communities. It has 1200 offices and psychological counseling centers nationwide. Emdad Relief Organization, Prisons and Corrective Measures Organization, Ministry of Youth and Sports, Ministry of Education, The Police Force and a variety of NGOs provide similar services.
5. According to Iran 2025 Vision, the country must strive for the health, welfare, nutritional safety, social security and access to equal opportunities and a healthy environment of its citizens.
6. The mental health program of 2012 was inaugurated by the Minister of Health. The program has defined, implemented and monitored mental health. The strategies for improving mental health is to update mental health services, reduce mental health risk factors by increasing intersectional cooperation and compare the performance of the provinces annually to ensure equality (21).
7. In 2015, the national mental health organizations were obliged to reduce risk factors and improve employee and student mental health by the High Council for Health and Food Security.
8. The Mental Health Act of Iran, in which the participation of 1700 NGOs and 144 city and village councils has been stressed, has been put on the agenda and will soon be ratified by the Parliament.
9. The provincial charter for health management and the joint communiqué of the Minister of Interior and the Minister of Health determine the pattern of cross-sectional and private participation.

***Mental Health Resources***

The mental health-related facilities and human resources are presented in Table 2. Mental health resources have been unbalanced and inequitable because their services have been provided without a pilot study.

Principles, Conceptual Model and Precedents 

After reviewing the resources and the comments of stakeholders, the ultimate goal of a promising mental health service was acknowledged an improvement in both social and mental health indices. Indices of greatest importance are as follow:

1. Reduce the incidence of mental disorders
2. Improve happiness index
3. Improve preventative social and mental behavior and mental health education
4. Improve mental health responsiveness
5. Increase financial support for mental patients
6. Improve indices for social health including social support

The socio-mental health services were based on the burden of disease survey in Iran and WHO recommendations (8, 9) and were designed according to a conceptual model. The services fall into 2 categories: Basic socio-mental health services and advanced socio-mental health services.

To determine expectations and tasks of social and mental health organizations, the High Council of Health and Food Security and the High Council of Districts should be the supportive resources and advocate intersectoral cooperation. 

The final model has 2 levels of health services: Basic and advanced mental health services. 

A.   Basic (level 1) services: These services include public education on the basics of social and mental health skills and social and mental health screening. At this level, cases with target mental conditions are recognized by community health workers and are referred for further evaluation and intervention to mental health specialists. A community health worker with at least 4 years training post high school education should be able to treat mood disorders and refer those with persistent psychosis and mood disorder to a specialist. They provide brief counseling for patients with a behavioral risk, train individuals with mental disorders and perform social evaluation. At the basic level, the health centers are the hub of the mental health system covering 12500 people. The mental health team for level 1 services includes 4 community health workers (one for every 3000 people), a socio-mental health expert and a general practitioner. 

Patients in need of social services should be referred to a relevant service provided by Welfare Organization, Emdad Relief Organization, employment offices, the Literacy Promotion Movement, employer syndicates, technical and vocational training organizations, charities and non-governmental organizations and other providers in order to strengthen their social support. 

B.   Advanced (level 2) services: The advanced socio-mental health services include facilities for mental and social emergencies and provide treatment for referrals from level one. This level includes treatment of psychosis and mood disorders with medication, advanced drug addiction services (psychotherapy, group therapy), hotlines for mobile intervention teams, crisis intervention (outpatient and short-term hospitalization) and post-discharge services (home follow-up, family education and patient education). These services should be delivered by a collaborative care team (including psychologists, clinical psychiatrists etc.) at the integrated mental health centers or clinics at the county level or by public or psychiatric hospitals.

## Discussion

This study suggests that it is necessary to provide socio-mental health services at 2 basic and advanced levels. The merging of social services with mental health services is because the evidence suggests a strong relationship between mental and behavioral disorders and the socioeconomic status of the patients. Poverty and low socioeconomic status are independent determinants of depression. 

Currently, there are many models for providing mental health services, but they might not be suitable for the context of this country. Therefore, it was necessary to redesign a local model suited for Iran’s context. The main reason was this model is based on the current structure of policymaking and executive bodies in the country. Moreover, motivation of all stakeholders was considered in designing this model. In addition, we tried to design a model compatible with WHO recommendations for a mental health system, (1) and our model embraces all 12 characteristics introduced by the WHO recommendation. 

The prerequisites of this model are as follow: 

1.   Defining a disciplinary relation between the health system and social emergency hot line of 123 

2.   Evaluating the existing human resources and training new staff to provide socio-mental health services in urban health centers 

**Table1:** Main Stakeholders of the Mental Health in Iran Addressed in This Study

**Type of Stakeholder**	**Those We Addressed**
**Managers of health system**	Three senior directors of provincial health authorities, AKA deputies of health at the universities of medical sciences and health services
**Experts **	WHO* Representative in Iran Secretariat of Health Policy, MOH** Iranian Academy of Medical Sciences Three faculties specialized at health systems research Three socio-mental health experts from the MOH
**Health care mediators**	Iranian Psychology Association Iranian Clinical Psychology Association Iranian Psychiatric Association Vice-President, High Councils of Provinces Psychology and Counseling Organization of Iran State Welfare Organization State prisons and security and Corrective Measures Organization Emdad Relief Organization Two related community-based NGOs Representatives of private hospitals

* WHO: World Health Organization

** MOH: Ministry of Health

**Table2:** Available Mental Health Facilities and Manpower

**Mental Health Facilities and Manpower**	**Number, Nationwide**
Number of psychiatric beds	9000 (with an inequitable distribution in the country)
Number of psychiatric emergency beds	248 (not available in 17 provinces)
Number of child psychiatry beds	229 (not available in 23 provinces)
Single-specialty psychiatric hospitals	34 (not available in 9 provinces)
General hospitals with psychiatric ward	76
Outpatient clinics	115
Psychiatric clinic	45
Private clinics	502
Psychiatrist	About 1,800 (41% settled in Tehran)
Clinical psychology practitioners	174
Trained general practitioners(GPs)	3,800
GPs with postgraduate psychiatry certificates	200
Health volunteers, all women	100,000
Community health workers and health technicians	5,000

Adapted from: Hajebi A, Damari B, et al. What to do to promote mental health of the society: A review on country’s mental health status and forthcoming strategies.2012. Iranian J Publ Health, Vol. 42, (Supple. No.1).

3.   The capacity of the private and non-profit sector for socio-mental issues must be considered for basic and advanced level services. 

4.   Implementing this model requires 4000 socio-mental health experts, 4000 urban health centers and 420 Community Mental Health Centers; these centers will generally be located at hospitals.

5.   To merge social and mental services, it is essential to have the authorization of the High Council of Health and Food Security and State Social Council.

6.   Evaluating, monitoring and modifying the model should also be considered. Intersectoral and provincial committees should set up the services for observation and data recording. 

This model is scheduled to be tested in a one-year pilot study in 3 districts later in 2016. The pilot study would provide enough evidence on efficiency and efficacy of this model.

## Limitations

The inability of the health system to control external factors affecting mental health and shortage of financial and human resources are the main challenges on the path to implement this model.

## Conclusion

The key features of this new model for providing mental health services in Iran, which is going to be tested in a pilot study in 2016, are setting up a system for organized referrals to specialized mental facilities and compatibility with the existing primary health care system. 
